# The physiology of forager hydration and variation among harvester ant (*Pogonomyrmex barbatus*) colonies in collective foraging behavior

**DOI:** 10.1038/s41598-019-41586-3

**Published:** 2019-03-26

**Authors:** Daniel A. Friedman, Michael J. Greene, Deborah M. Gordon

**Affiliations:** 1 Department of Biology, Stanford University, Stanford, California, USA; 2 Department of Integrative Biology, University of Colorado Denver, Denver, Colorado, USA

## Abstract

Ants are abundant in desiccating environments despite their high surface area to volume ratios and exposure to harsh conditions outside the nest. Red harvester ant (*Pogonomyrmex barbatus*) colonies must spend water to obtain water: colonies lose water as workers forage outside the nest, and gain water metabolically through seeds collected in foraging trips. Here we present field experiments showing that hydrated *P. barbatus* foragers made more foraging trips than unhydrated nestmates. The positive effect of hydration on foraging activity is stronger as the risk of desiccation increases. Desiccation tests showed that foragers of colonies that reduce foraging in dry conditions are more sensitive to water loss, losing water and motor coordination more rapidly in desiccating conditions, than foragers of colonies that do not reduce foraging in dry conditions. Desiccation tolerance is also associated with colony reproductive success. Surprisingly, foragers that are more sensitive to water loss are from colonies more likely to produce offspring colonies. This could be because the foragers of these colonies conserve water with a more cautious response to desiccation risk. An ant’s hydration status may influence its response to the olfactory interactions that regulate its decision to leave the nest to forage. Thus variation among ant colonies in worker physiology and response to ambient conditions may contribute to ecologically significant differences among colonies in collective behavior.

## Introduction

Animal behavior and physiology are jointly shaped over evolutionary time by environmental conditions. For terrestrial animals, desiccation is among the most important physiological stressors^1–3^. Many animals, such as horses^4^, frogs^5^, and beetles^6^, exhibit behavioral preferences that minimize exposure to desiccating conditions. Insects are successful and diverse in desiccating environments, despite their small size which leads to a high surface area compared to body volume^1,2,7–15^. In such arid conditions, insects demonstrate behavioral^16–19^ and physiological^20–24^ adaptations. In social insects, selective pressures from abiotic environmental stressors result in adaptations to worker physiology, and also in the collective behavior arising from interactions among workers. The behavioral ecophysiology of hydration has been well studied in desert ants, whose colonies face significant desiccation stress, especially when foraging outside the nest^25–32^. Here we examine how variation among colonies in desiccation physiology can shape the collective behavior that regulates foraging activity.

Red harvester ant (*Pogonomyrmex barbatus*) colonies gain water through the oxidation of fats from seeds they eat^33^, and lose water primarily by cuticular evapotranspiration^25,34,35^. Thus colonies must spend water to obtain water: foragers lose water as they forage, but gain water in the form of seeds. To manage this tradeoff between water loss and obtaining food and water, foraging activity is regulated through interactions among workers inside the nest^36–40^. Desert harvester ant species regulate colony foraging activity in response to changes in many factors, including humidity^37^, temperature^41–43^, and predation pressure^44^. In *P. barbatus*, outgoing foragers use the rate at which they meet returning foragers inside the nest to decide whether to leave the nest on the next foraging trip^38–40^. Interactions inside the nest are olfactory, based on brief antennal contacts in which one worker assesses the cuticular hydrocarbon profile of another and the odor of seeds^36,38^. When food availability is high, foragers tend to find food more quickly and to return at a higher rate, thus stimulating more foragers to leave the nest to forage^40,45^. Foraging ends at about mid-day in the summer, as temperature increases and humidity decreases through the morning and into the afternoon^42,46^. It appears that an outgoing forager’s estimate of ambient humidity, from previous foraging trips, influences its response to interactions with returning foragers and its decision to leave the nest on its next trip^47^.

Natural selection is shaping the regulation of foraging activity in harvester ants^48^. A colony lives for 20–30 years, as long as the founding queen survives to produce workers. Adult colonies differ in how they regulate foraging in hot, dry conditions^37,49,50^. Only about 25% of colonies succeed in producing offspring colonies^51^, and variation among colonies in reproductive success is associated with variation in the regulation of foraging activity. Colonies that tend to forage less in dry conditions and show more stable foraging in humid conditions, are more likely to produce offspring colonies^48^. Transcriptomic studies of forager brains show gene expression differences between two sets of *P. barbatus* colonies that differed in whether they reduce foraging in dry conditions^52^. This suggests that differences among colonies in the collective regulation of foraging activity are associated with differences in worker neurophysiology.

Here we investigated the relationship between red harvester ant colony foraging activity and forager water content and water loss rate to understand how behavioral decisions by ants are affected by desiccating conditions. First, we tested how a forager’s water hydration status influences its decision to leave the nest to forage in conditions that vary in risk of water loss. Next, we compared water content, rate of water loss, and the effect of water loss on survival in colonies that differed in the regulation of foraging activity and reproductive success.

## Materials and Methods

### Study site

Experiments were conducted at the site of a long-term study of a population of about 300 colonies near Rodeo, NM, USA (GPS: 31.8700, −109.0389)^51^. All colonies used in experiments were mature colonies of at least 5 years of age (colony ages known from an annual census)^51^. Our study was performed during the summer. Colonies *of P. barbatus* colonies are not active during the winter, and we do not consider seasonal dynamics here.

### Effect of forager hydration on foraging activity

To test whether an ant’s decision to leave the nest to forage depends on its hydration level, we collected foragers, manipulated their hydration levels, returned them to their nest, and observed their foraging behavior the following day. Experiments were performed with five adult colonies of *P. barbatus* adjacent to the long-term study site. Two colonies were observed on 8/18/2016, two colonies were observed on 8/20/2016, and one colony was observed on 8/25/2016.

On Day 1 of the experiment, foragers were collected with an aspirator between 6:30am and 8:00am, early in the morning activity period^53^. An ant was identified as a forager when it was on a foraging trail or fan, more than 1 meter from the nest entrance, either walking away from the nest carrying nothing or walking towards the nest with a seed^45^. Foragers of *P. barbatus* do not perform other tasks; and workers collected when foraging are unlikely to perform other tasks the next day^53^. Ants were anesthetized by cold, and marked with spots of oil paint on their head (Uni-Paint G0538596) according to treatment group; colors were rotated each day. The ant was placed on its side to avoid paint sticking to the container; by the time the ant was mobile the paint was dry. Marked ants do not appear to act differently from unmarked ants, and are rarely rejected by nestmates^53,54^. The number of ants per treatment was matched within a colony for each experiment, and ranged from between 130–150 ants per group (Table 1). Ants in the unhydrated treatment group were marked without being administered water. Ants in the hydrated treatment group were marked with a different color of paint, and administered 0.2 μL of pure water (Millipore). For ants in the hydrated treatment group, the droplet of water was placed in between their mandibles. The surface tension of a droplet of water breaks when it touches the notched clypeus from the top, or when it contacts the bristles under the mandibles. Ants did not drink the entire droplet, but previous work shows that solutions administered this way are at least partially consumed by workers^52^. Ants were returned to their nest during the afternoon of Day 1.

**Table 1:** Experimental design summary for hydration experiment.

Colony	Ants per treatment group	Hydrated foraging trips	Unhydrated foraging trips	Overall ratio (hydrated/unhydrated)
D37	130	383	327	1.17
D22	130	319	262	1.22
D11	140	603	519	1.16
N5	140	875	764	1.15
D40	150	1171	1013	1.16

The “Overall ratio” is the total number of foraging trips made by hydrated ants divided by the total number of foraging trips made by unhydrated ants of the same colony on a given day.

On Day 2, we observed the foraging behavior of marked ants from focal colonies. Data collection began shortly after sunrise, when the first marked ant was observed to initiate a foraging trip. An outgoing foraging trip by a marked ant was recorded when it walked off the nest mound in a direction taken by other foragers. During the colony’s entire morning foraging activity period, we recorded foraging trips made by marked ants and summarized the counts in 30-second intervals. On the two days when experiments were performed for two colonies (8/18 and 8/20), each colony was observed for alternating periods of 20–25 minutes (40–50 sequential 30-second intervals). The average duration of a *P. barbatus* foraging trip is about 20 minutes^55,56^, and a forager spends some time inside the nest between trips^56^. Thus it is unlikely that any marked ant made more than one foraging trip during each 20–25 minute observation period. On the day when a single colony was observed (8/25/16), observations were continuous. Observation of each focal colony continued until there was a 3-minute interval in which no marked ants left the nest to forage, at the end of that colony’s morning activity period.

We used R v3.5.1^57^ (script provided) to statistically analyze the effect of hydration on foraging activity. We compared the total number of foraging trips made by hydrated ants to the number of trips made by the same number of nestmate unhydrated ants, using a paired sample Wilcoxon signed-rank test. To test whether differences between the treatment groups were consistent throughout the day, the foraging activity period of each colony was partitioned into three intervals, each with an equal number of observation periods, and we compared in each interval the number of foraging trips made by hydrated to the number of trips made by unhydrated ants, using a paired sample Wilcoxon signed-rank test. All foraging data from the 5 colonies over the 3 days were included in this analysis.

We examined how manipulation of hydration status influenced a forager’s response to changes throughout the day in temperature and humidity. Temperature and relative humidity values at the field site were recorded every 30 seconds using an iButton (Maxim Integrated, San Jose, California, USA) placed on the surface of the ground away from direct sunlight. The temperature and humidity readings were averaged for each interval in which marked ants were observed. After removing early-morning observation periods for which temperature and humidity data were not recorded (N = 0–2 per/colony), there were 25 observation periods of 20–25 minutes each included in the analysis (5 colonies, 3 to 7 periods from each colony). For each observation period, we calculated the ratio of the total number of foraging trips made by hydrated ants to the total number of foraging trips made by unhydrated ants. Temperature increases and humidity decreases in the course of the morning activity period^47^, and thus combine to increase desiccation stress. To account for this, temperature and humidity recordings for each of the 25 observation periods were combined as per Allen *et al*.^58^ to calculate a Vapor Pressure Deficit (VPD) statistic. Higher VPD values reflect higher rates of water loss.

A linear regression was performed with the ratio of hydrated to unhydrated foraging trips as the dependent variable and VPD as the independent variable. A quadratic regression was used to fit VPD as the independent variable to the ratio of hydrated to unhydrated foraging trips as the dependent variable. Models were compared for goodness of fit using the Akaike information criterion^59^ and an ANOVA model test. To test for colony-specific effects of hydration, we performed the same linear and quadratic regressions as above, with colony identity added as a random categorical factor in the model specification. Our experiment was not specifically designed to parameterize each colony’s sensitivity to hydration, because each of the 5 colonies was measured only once, conflating the effect of differences among days in the effect of hydration with the plausible effect of differences among colonies in sensitivity to hydration.

### Forager sensitivity to water loss and variation in colony behavior

We used desiccation experiments to compare the physiological characteristics of N = 74 foragers from N = 24 colonies. Foragers were collected from all 24 colonies in August 2014. 1 to 4 foragers were collected from each colony by hand (wearing gloves), and used in experiments within 4 hours. We classified the 24 colonies in two ways. First, we classified colonies into two groups according to reproductive success in offspring colonies: (1) colonies that were found to have offspring colonies in a previous parentage analysis^51^ (N = 12) and (2) colonies that were not found to have offspring colonies (N = 12 colonies). Second, we classified colonies into two groups according to whether they reduced foraging activity in response to dry conditions^37,48,50,60^. “High Foraging” colonies (N = 6) did not reduce foraging activity in dry conditions, “Low Foraging” colonies (N = 10) significantly reduced foraging activity in dry conditions. Of the 23 colonies, there were 8 colonies for which foraging response to dry conditions had not been measured. As previous work showed^48^, the colonies that had offspring tended to be the ones that reduce foraging activity in dry conditions.

To explore the relation among water loss physiology, foraging activity in dry conditions and colony reproductive success, we performed three analyses in R v3.5.1^57^ (script provided). First, we tested how forager water loss physiology was associated with colony reproductive success in offspring colonies. Second, we tested how forager water loss physiology was associated with whether a colony reduces foraging in dry conditions.

To measure forager water loss physiology, we inferred rates of water loss based upon decrease in body mass in desiccating conditions^25,27,32^. Water loss measurements were made in the Technical Equipment Laboratory at the Southwestern Research Station, Portal, Arizona USA. The initial body mass of each live ant was measured to the 0.1 ug using a Mettler AT261 Delta Range balance. Ants were then placed into 1.5 ml Eppendorf vials that had been modified with eight 2 mm diameter holes to allow for air exchange, and labeled with a coded number on the cap. Vials were then placed on a 2 cm deep bed of anhydrous calcium sulfate desiccant (Drierite, 8 mesh) in a metal tray that was placed in a 40 degree Celsius oven (Napco Model 320). The desiccant was added to lower the relative humidity of the air around the ants as much as possible. A data logger was placed with the samples to measure relative humidity and temperature (HOBO, Onset Computer, Bourne, MA, U.S.A.). The data logger was too large to place into an Eppendorf tube, but was placed at a similar depth in the desiccant as the perforated Eppendorf tubes containing ants. Every 20 minutes, we observed the ants in the vials and recorded the mass of each ant and the time when the ant was morbid, defined as being unable to right itself when placed on its back, and when it was dead, using techniques in^2,23,61^. All measurements were conducted in a blinded fashion; as the identity of each ant was coded, the observer did not know the identity of any individual ant. A dry mass was calculated after death; the ants were dried at 60 degrees Celsius until dry mass measurements did not change after two measurements separated by 2 hours. The water loss measurements were performed in two trials on two separate days, with an approximately equal number of foragers run on each day from each group of colonies. On day 1 of the experiment, temperature was maintained at 41.1 degrees +/− 1.23 S.D. Celsius and 5.57% +/− 1.36 S.D. relative humidity. On day 2, temperature was maintained at 41.2 degrees +/− 1.30 S.D. Celsius and 21.0% +/− 2.0 relative humidity. The higher humidity on day 2 was a result of a rainstorm that increased ambient air humidity in the laboratory. In our statistical analyses, we account for the difference between the two days in relative humidity by standardizing our mass measurements by comparing gross cuticular permeabilities in units of mg cm^−2^ torr^−1^ as per previous work^2,28,62^.

We calculated mass loss rate by dividing the difference between live body mass (mg) and moribund mass (mg) by the duration of time between the start of the experiment and the time at which moribund mass was taken^25,27,32^.The total water content (TWC) is the percentage of starting body mass that was due to water. The TWC of each ant was calculated as a percentage of live body mass using the following equation: TWC (%) = ((Live Mass – Dry Mass) x 100)/Live Mass. The critical water content (CWC) is the percentage of body mass lost due to water loss when an ant becomes moribund and cannot walk or right itself. CWC was calculated as the percentage live mass lost by water loss at the point moribund mass was measured: CWC (%) = ((Moribund Mass − Dead Mass) × 100)/Live Mass. We estimated the surface area of ants using the following equation, created for a congener *P. rugosus*^25^ that, like *P. barbatus*, has monomorphic workers: Surface area = 0.103 × Mass^0.667^. Gross cuticular permeability is the water loss experienced by an ant accounting for both an ant’s surface area and the vapor pressure surrounding the ant. Gross cuticular permeability (mg cm^−2^ hr^−1^ Torr^−1^) was calculated as [(Total Water Loss Rate (mg hr^−1^)/Surface area (cm^2^))/Water Pressure Saturation Deficit (Torr)]. Area-independent water loss rate was calculated in units of ug/hr/cm^2^ by dividing water loss rate to a state of morbidity by the surface area of an ant. Water pressure saturation deficit was calculated as per the CRC handbook^63^.

We examined five measures of forager water loss physiology: Total water content, Critical water content, Time to morbidity, Time to death, and Area-independent rate of water loss. To test for an association between forager water loss physiology and colony reproductive success, we used a Wilcoxon signed rank test to compare colonies that did (N = 12) versus did not (N = 12) have offspring colonies for all of the five measures. To test for an association between forager water loss physiology and the regulation of foraging activity, we performed a Chi-square test for differences among three groups of colonies (High foraging; did not reduce foraging in dry conditions N = 6, Low foraging; reduced foraging in dry conditions N = 10, Foraging activity not measured N = 8) in all of the five measures. To test for differences in all five measures between each pair of these 3 groups of colonies that differed in the regulation of foraging, we used a Wilcoxon signed rank test. We used Principle Component Analysis using the “factoextra” package^64^ to examine how forager water loss physiology was associated colony reproductive success and whether a colony reduces foraging in dry conditions. The PCA was performed on 5 variables, the same 5 measures as above: Total water content, Critical water content, Time to morbidity, Time to death, and Area-independent rate of water loss.”

## Results

### Effect of forager hydration on foraging activity

Hydrated ants made more foraging trips than unhydrated ants (Table 1). Pooling data from all colonies, hydrated ants made 1.18-fold more foraging trips than their unhydrated nestmates (3125 trips for hydrated versus 2652 trips for unhydrated ants from 5 colonies, paired sample Wilcoxon signed-rank test, p = 0.0006, Table 1).

**Figure 1:**
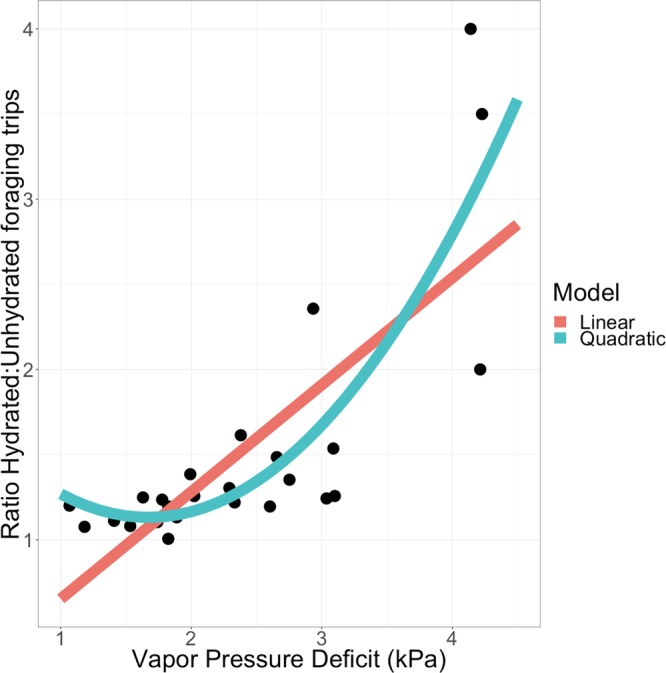
Effect of Vapor Pressure Deficit on the effect of hydration on foraging. The X axis is Vapor Pressure Deficit in kilopascals (kPa). Higher values correspond to more desiccating conditions. The Y axis is the ratio of foraging trips of hydrated to unhydrated nestmates during each observation period (N = 25 periods across N = 5 colonies). Regression models are plotted for linear (red) and quadratic (blue) fits.

The more desiccating the conditions, with higher Vapor Pressure Deficit (VPD), the more likely were hydrated ants to make more foraging trips relative to unhydrated ants (Fig. 1, linear regression p < 0.001, r^2^ = 0.564). A quadratic model provided a better fit, measured by proportion of variance explained (Fig. 1, p < 0.001, r^2^ = 0.688), and also through direct model comparison (difference in AIC = 7.514 favoring the quadratic model, ANOVA p = 0.0042). This supports a non-linear effect of VPD on the influence of hydration on foraging trips.

When colony identity was considered as a categorical factor, VPD remains significantly associated with the effect of hydration on foraging (quadratic regression, p < 0.001, r^2^ = 0.560), but colonies do not significantly vary in how changes in humidity influence the effect of hydration (all colonies with effect p > 0.05). The quadratic regression model with colony-specific effects was not more informative than a quadratic model that does not consider colony identity (ANOVA for Colony*Humidity term p > 0.8, difference in AIC = 10.947 favoring the model without colony). Hydration experiments with replicated measurements for each colony would be needed to determine whether colonies differ in their sensitivity to hydration.

Hydration increased foraging activity most when humidity was low, later in the morning foraging activity period (Table 2). During later two thirds of the foraging activity period, between about 8:30 a.m to noon, when VPD increases toward its midday high, hydrated ants made significantly more foraging trips than unhydrated ants (1.29-fold increase in trips by hydrated ants, Wilcoxon signed-rank test, p < 0.001). However, during the earliest third of the colony’s foraging period, at around 7am to 8:30 a.m., when humidity is highest and VPD is lowest, there was no significant difference between the number of foraging trips made by hydrated and unhydrated foragers of a given colony (1.03-fold increase in trips by hydrated ants, Wilcoxon signed-rank test, p = 0.62).

**Table 2:** Number of foraging trips made by hydrated and unhydrated ants in each third of the morning foraging activity period.

Colony	Section	Hydrated trips	Unhydrated trips
D37	1	192	191
	2	142	113
	3	49	23
N5	1	223	231
	2	309	264
	3	343	269
D11	1	156	138
	2	117	90
	3	104	58
D22	1	103	105
	2	116	97
	3	100	60
D40	1	478	457
	2	435	353
	3	258	203

### Water loss and colony differences in behavior

**Figure 2:**
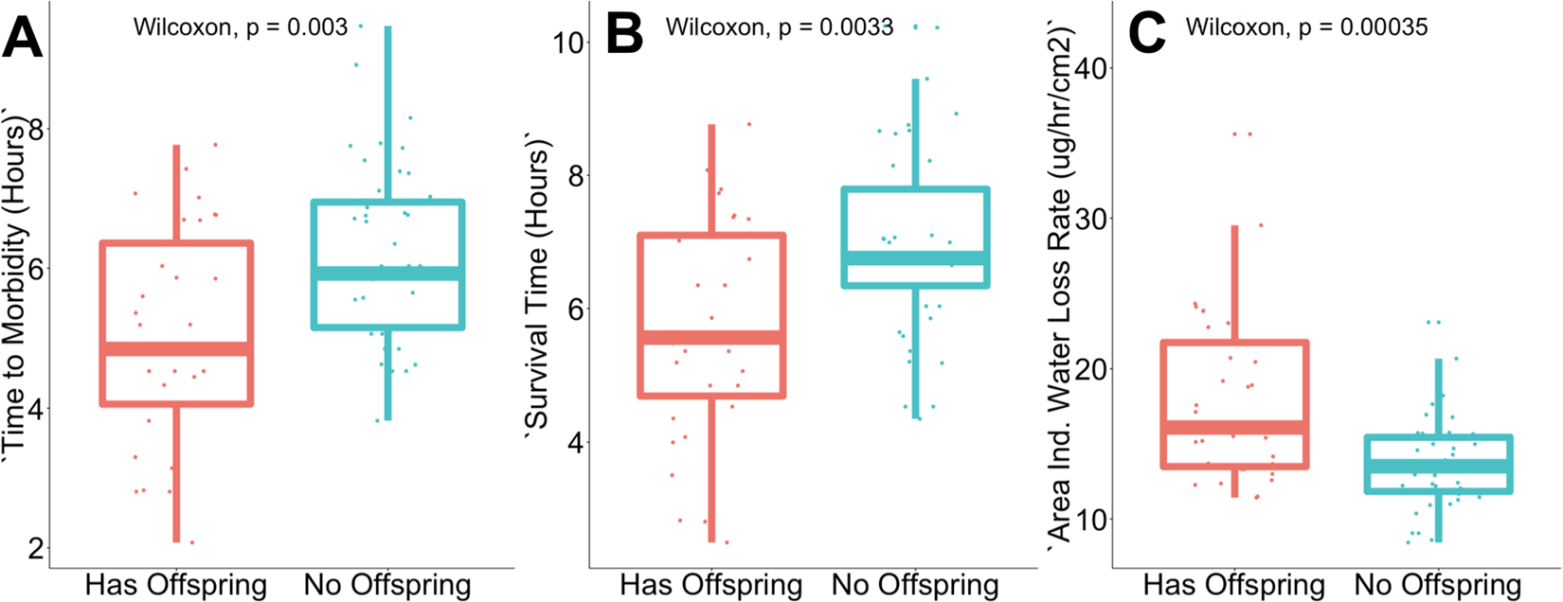
Differences in colony reproductive success in offspring colonies, and forager time to (**A**) morbidity, (**B**) survival time, and (**C**) water loss rate. Box plot reflects the median and 25^th^ and 75^th^ percentile of each distribution.

Colonies with higher reproductive success, that had offspring colonies, had lower desiccation tolerance (Fig. 2). Foragers from colonies that had offspring colonies reached morbidity (Fig. 2A, Wilcoxon test, p = 0.003, W = 5.98, r_Spearman_ = 0.35) and death (Fig. 2B, Wilcoxon test, p = 0.0033, W = 5.99, r_Spearman_ = 0.34) significantly more rapidly than foragers from colonies without offspring colonies. Foragers from colonies that had offspring colonies lost water at a significantly faster fate (Fig. 2C, Wilcoxon test, p = 0.00035, W = 6.90, r_Spearman_ = −0.42) than colonies without offspring colonies, but did not differ in total water content (Wilcoxon test p = 0.59) or critical water content (Wilcoxon test p = 0.43).

**Figure 3:**
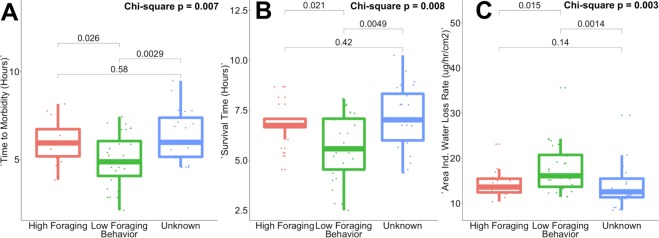
Differences in the regulation of foraging in dry conditions, associated with time to (**A**) morbidity, (**B**) survival time and (**C**) water loss rate.

Whether a colony reduces foraging activity in dry conditions was significantly associated with forager time to morbidity (Fig. 3A, Chi-square, p = 0.007, χ^2^(2) = 10.02), time to death (Fig. 3B, Chi-square, p = 0.008, χ^2^(2) = 9.76), and average water loss rate (Fig. 3C, Chi-square, p = 0.002, χ^2^(2) = 12.63). Colonies that reduce foraging in dry conditions had lower desiccation tolerance compared to colonies that do not reduce foraging in dry conditions. Foragers from colonies that reduced foraging in dry conditions tended to become morbid (Fig. 3A, Wilcoxon test, p = 0.026, W = 3.17) and die (Fig. 3B, Wilcoxon test, p = 0.021, W = 3.28) more quickly than foragers from colonies that did not reduce foraging in dry conditions. In addition, foragers from colonies that reduced foraging in dry conditions lost water at a higher rate than foragers from colonies that did not reduce foraging in dry conditions (Fig. 3C, Wilcoxon test, p = 0.015, W = 3.46). Low and high foraging colonies did not differ in critical water content (Wilcoxon test, p = 0.371) or total water content (Wilcoxon test, p = 0.898, W = 0.181).

We further compared each of the two groups of colonies whose foraging activity had been measured (did or did not reduce foraging in dry conditions) with the 8 colonies, none of which had offspring colonies, for which foraging activity was not measured. Foragers from colonies that reduced foraging in dry conditions more rapidly reached morbidity (Wilcoxon test, p = 0.003, W = 4.43) and death (Wilcoxon test, p = 0.0049, W = 3.99) than the 8 colonies whose foraging activity was not measured. Foragers from colonies that reduced foraging in dry conditions also lost water at a significantly higher rate than those from colonies with no offspring whose foraging activity was not measured (Wilcoxon test, p = 0.0014, W = −4.52). Colonies whose foraging activity was not measured did not significantly differ from colonies that did not reduce foraging in dry conditions in time to morbidity (Wilcoxon test, p = 0.58), time to survival (Wilcoxon test, p = 0.42), or water loss rate (Wilcoxon test, p = 0.14).These results are consistent with the hypothesis, supported by the association found in previous work^48^, that the 8 colonies whose foraging behavior was not measured but had no offspring were likely to be “High Foraging” colonies that do not reduce foraging activity in dry conditions.

**Figure 4:**
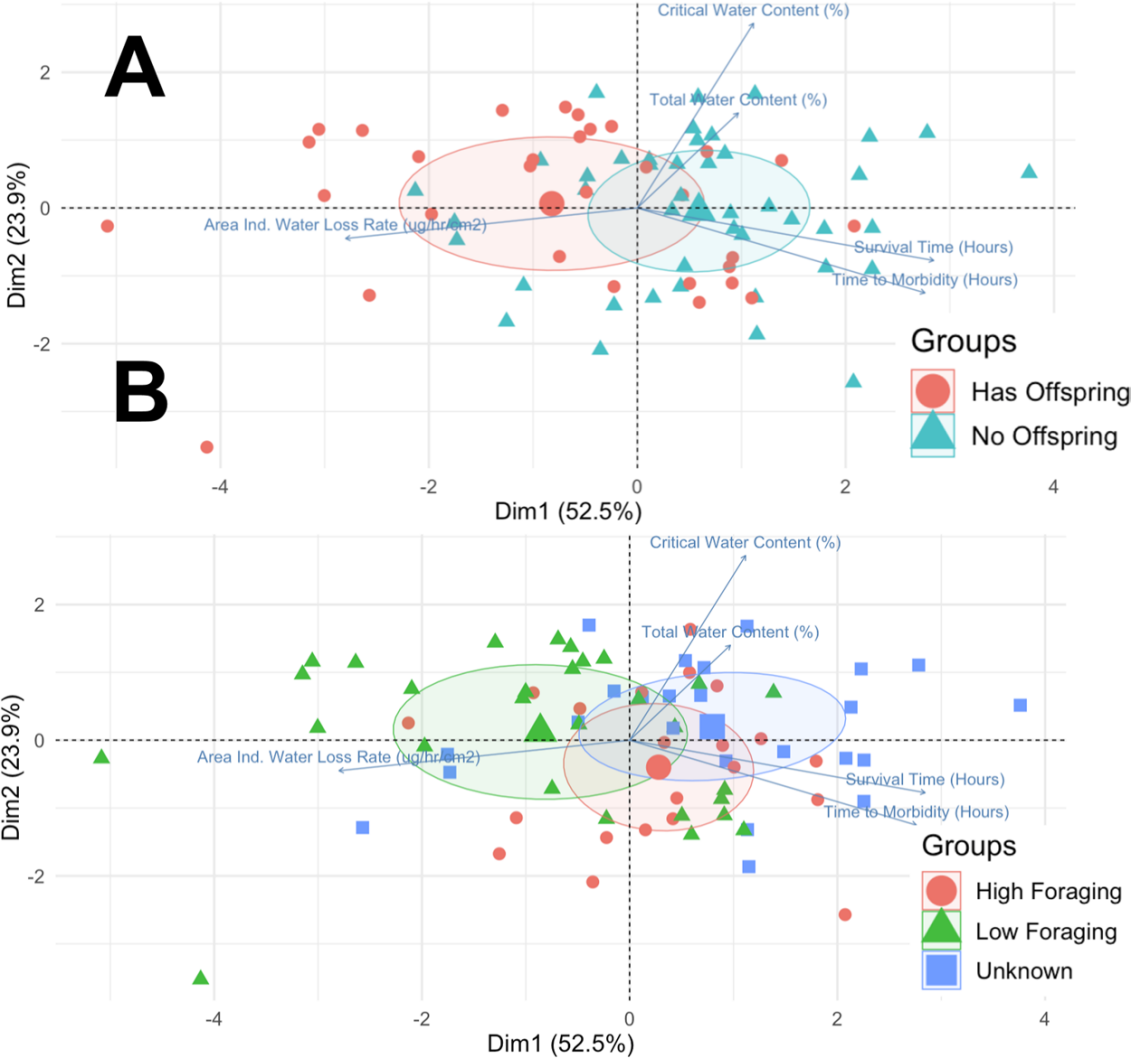
PCA projection of variables related to desiccation physiology. Each point represents data from a single ant (N = 74) Arrows labeled with variable names show the loading of each physiological measurement along the PC axes. (**A**) Color corresponds to colony reproductive success, either has offspring (red) (N = 12 colonies) or no offspring (blue) (N = 12 colonies). (**B**) Color corresponds to colony regulation of foraging in dry conditions: High foraging in dry conditions (red) (N = 6 colonies), Low foraging in dry conditions (green) (N = 10 cols). Blue shows data from the 8 colonies for which no foraging data were available. Each shaded ellipse outlines the oval that captures 30% of the points for each group.

Principal Component Analysis showed that both the regulation of foraging activity and colony reproductive success are associated with desiccation physiology. Colonies that reduce foraging in dry conditions had higher water loss rates, and shorter times to morbidity and death in desiccating conditions, than colonies that do not reduce foraging in dry conditions (Fig. 4). PC1 captured 52.5% of the variation among samples and shows the negative correlation between time to morbidity/death and water loss rate. Samples from colonies that reduced foraging in dry conditions were further towards the left “high rate of water loss” end of PC1 compared to the group of colonies that did not reduce foraging in dry conditions and the group of colonies whose foraging activity was not measured (Fig. 4A). Rate of water loss, and time to morbidity and death in desiccating conditions, were also related to colony reproductive success. Colonies with and without offspring differ along the PC1 axis, related to water loss and survival, but not at all on the PC2 axes, related to water content. PC2 captures 23.9% of the variation among samples and is associated with increases in total and critical water content. axis.

## Discussion

Desiccation is among the most important stressors for terrestrial animals. For desert ants, desiccation is an important abiotic stressor that shapes the evolution of forager behavior and physiology in desert ants. Here we show that a harvester ant forager’s hydration level influences the probability that it leaves the nest to forage. The positive effect of hydration on foraging activity is stronger as the risk of desiccation increases (Fig. 1). Colonies differ in both the desiccation tolerance of their foragers and in how they adjust foraging activity to dry conditions. Desiccation tests showed that in colonies that reduce foraging in dry conditions, foragers are especially sensitive to water loss. Foragers from colonies that reduce foraging in dry conditions lose water and motor coordination more rapidly than foragers from colonies that do not reduce foraging in dry conditions (Fig. 2). Desiccation tolerance is also associated with colony reproductive success (Figs 3 and 4). Surprisingly, colonies that are more sensitive to water loss are more likely to produce offspring colonies, apparently because their foragers regulate foraging more closely in response to desiccation risk and thus conserve water.

The results presented here suggest that an outgoing forager’s decision to leave the nest is influenced by its state of hydration. This could explain how colony foraging activity is tuned throughout the day in response to changing ambient conditions^47^. Previous work shows that an ant’s decision to leave the nest depends on its rate of olfactory interactions (brief antennal contact in which one ant assesses the cuticular hydrocarbons of a nestmate)^36^ with returning foragers^39,40^; creating feedback that links foraging activity to food availability. Our result, that more hydrated foragers are more likely to leave the nest, indicates that an ant’s threshold response to olfactory interactions is influenced by its current hydration level.

Foragers from colonies that reduce foraging in response to desiccation risk are especially sensitive to desiccation, losing water and motor coordination faster than foragers from colonies that do not reduce foraging in dry conditions. Because of higher water loss rates, workers from colonies that reduce foraging in dry conditions reached morbidity and death significantly faster than workers from colonies that do not reduce foraging in dry conditions. However, colonies that differ in foraging behavior did not show differences in total water content or critical water content. Thus, differences among colonies in desiccation tolerance do not appear to be due to differences in initial water balance, but instead to differences in water loss rates across the cuticle. This suggests that forager sensitivity to desiccation may be a proximate individual-level mechanism underlying collective behavioral differences among colonies.

High sensitivity to desiccation in workers is associated with higher colony reproductive success in offspring colonies. Several mechanisms might explain the surprising result that colonies with higher reproductive success tend to have foragers that are more sensitive to desiccation. One possibility is that if a forager’s desiccation sensitivity influences its decision to forage in dry conditions, the more sensitive colonies would be likely to regulate foraging so as to conserve water, because their foragers are less likely to risk desiccation by leaving the nest to forage. This may lead to the association between reduced foraging in dry conditions and colony reproductive success^48^. In *Pogonomyrmex* and other desert ant species^16,34,65,66^, foundresses lose water rapidly due to cuticular abrasion while digging^29,31^, and rely on the first cohort of workers to restore their hydration and nutrition levels. The relation between the regulation of foraging and susceptibility to water loss may be especially important for young, small colonies. Small founding colonies that are more susceptible to water stress may be especially likely to avoid foraging in dry conditions. Further work is needed to learn whether avoiding water loss promotes survival despite the reduced food intake.

Selective pressures from the environment over evolutionary time shape physiological and behavioral phenotypes. In the social insects, colonies respond to changing conditions through collective behavior. Natural selection on collective behavior, as on any other phenotypic trait, is due to ecological pressures^67,68^. Since collective behavior arises from interactions among individuals, the target of selection is the way that interactions generate the behavior. To learn how selection shapes colony behavior response to environmental stressors, we can ask how worker physiology influences the interactions among workers that regulate colony activity.

### Ethics

No permits, special permissions, or institutional animal care and use protocols were required to conduct the research. Use of animals in the study conforms with the Association for the Study of Animal Behaviour/Animal Behavior Society Guidelines for the Use of Animals in Research (*Animal Behaviour*, 2006, 71, 245-251).

## Supplementary information

Supplementary Dataset 1

Supplementary Dataset 2

## Data Availability

All datasets and R scripts used for statistical analysis are included as Supplementary Files.
